# Integrating radiosensitivity index and triple‐negative breast cancer subtypes reveals SERPINB5 as a radioresistance biomarker in triple‐negative breast cancer

**DOI:** 10.1002/ctm2.1787

**Published:** 2024-08-07

**Authors:** Xinxin Rao, Xuanyi Wang, Chao He, YiZhou Jiang, ZhiMing Shao, Yan Feng, Jundong Zhou, Xiaomao Guo, Xingxing Chen

**Affiliations:** ^1^ Department of Radiation Oncology Fudan University Shanghai Cancer Center Shanghai China; ^2^ Department of Oncology Shanghai Medical College, Fudan University Shanghai China; ^3^ Shanghai Clinical Research Center for Radiation Oncology Shanghai Key Laboratory of Radiation Oncology Shanghai China; ^4^ Suzhou Cancer Center Core Laboratory Nanjing Medical University Affiliated Suzhou Hospital Suzhou China; ^5^ Department of Breast Surgery Fudan University Shanghai Cancer Center; Key Laboratory of Breast Cancer in Shanghai Shanghai China; ^6^ Department of Radiation Oncology Nanjing Medical University Affiliated Suzhou Hospital Suzhou China

Dear Editor,

The heterogeneous nature of triple‐negative breast cancer (TNBC) necessitates the development of predictive biomarkers for radiotherapy (RT) outcomes. Our study integrates the Radiosensitivity Index (RSI) with the Fudan University Shanghai Cancer Center (FUSCC) TNBC classification^1^ to explore TNBC radiosensitivity. We identified a specific TNBC subpopulation—the integrated basal‐like immune‐suppressed (BLIS) and radioresistant (RR) subtype—exhibiting pronounced radioresistance and an increased risk of local recurrence post‐RT. Elevated expression of the SERPINB5 gene in this high‐risk group suggests its potential as a therapeutic target for radioresistance, advancing personalized RT strategies for high‐risk TNBC patients.

Our study involved 160 patients with pathologically confirmed TNBC (Table S1). Over a median 48‐month follow‐up, 22.5% of these patients experienced disease recurrence (DR). Patients with the RR subtype who received RT had a higher DR rate than those with the radiosensitive (RS) subtype (*p *= .0429; Figure 1A). RSI did not predict DR in TNBC patients who did not undergo RT (*p *= .33; Figure 1B). The interaction between RSI and RT was statistically significant (RSI x RT, *p *= .033), supporting RSI's specificity as a radiation‐specific signature, as previously reported.^2^, ^3^ Similar results were observed in public TNBC datasets from TCGA and METABRIC, further supporting our study's findings (Figure 1C,D and Figure S1A,B).

**FIGURE 1:**
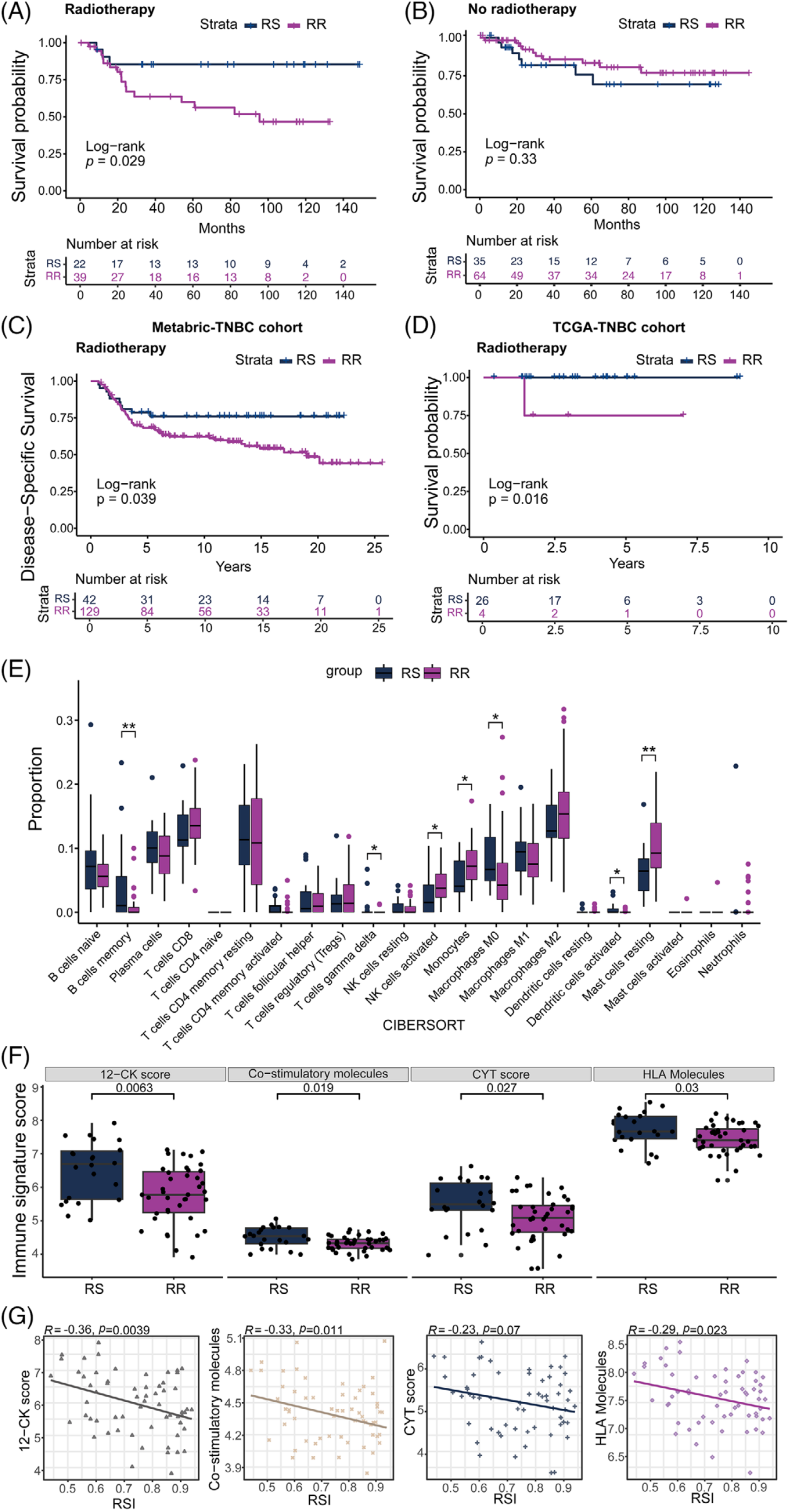
Association of the radiosensitivity index (RSI) signature with survival outcomes and immune repression. (A) Kaplan‐Meier plot illustrating recurrence‐free survival (RFS) differences between radiosensitive (RS) and radioresistant (RR) patients treated with RT. (B) Kaplan‐Meier plot showing RFS comparison between RS and RR patients without RT. (C) Kaplan‐Meier plot illustrating RFS differences between RS and RR patients treated with RT in the Metabric triple‐negative breast cancer (TNBC) cohort. (D) Kaplan‐Meier plot illustrating RFS differences between RS and RR patients treated with RT in the TCGA TNBC cohort. (E) Box plot depicting the proportion of immune cell infiltration in RS versus RR tumors in RT‐treated patients. ** indicates .001 < *p* < .01, while * represents .01 < *p* < .05. (F) Box plot illustrating differences in 12‐chemokine (12‐CK), co‐stimulatory molecules, cytolytic activity (CYT), and HLA molecules scores between RS and RR patients. (G) Dot plot demonstrating the correlation between RSI and scores of 12‐CK, co‐stimulatory molecules, CYT and HLA molecules.

Our analysis revealed distinct immunological profiles in RT‐treated TNBC patients with RR and RS tumors. Specifically, RR tumors showed increased immunosuppressive monocytes and resting mast cells, but decreased gamma delta T cells, activated dendritic cells, and B memory cells, compared to RS tumors (Figure 1E). They also had significantly lower scores in cytolytic immune markers, including 12‐chemokine (12‐CK), co‐stimulatory molecules, cytolytic activity (CYT), and HLA molecules (Figure 1F). Spearman's analysis showed a notable inverse relationship between RSI and evaluated immune scores (Figure 1G and Figure S1C–G), consistent with previous research linking RSI‐based tumor radioresistance and immune suppression in TNBC.^4^, ^5^

We examined the transcriptomic profiles of RT‐treated TNBC patients with and without DR. Hierarchical clustering primarily segregated the profiles of RT‐treated TNBC patients with DR from those without (Figure 2A). The analysis identified 458 differentially expressed genes in patients with recurrence, with 286 upregulated and 172 downregulated (Figure 2B). qRT‐PCR validation was conducted on eight genes implicated in tumor recurrence (Figure S2). Gene Ontology enrichment analysis revealed the downregulation of several immune‐related signaling pathways in patients with recurrence (Figure 2C). Additionally, dysregulation in genes related to cell proliferation, metabolism, tumor microenvironment, and immune response was observed (Figure 2D). Gene set enrichment analysis highlighted a systematic downregulation in immune response‐related gene sets in recurrent cases (Figures 2E,F). The CIBERSORT algorithm revealed distinct immune cell infiltration landscapes in normal versus tumor tissues of RT‐treated TNBC patients (Figure S3A), with tumor tissues showing fewer cytotoxic CD8 T cells and more immunosuppressive M2 macrophages compared to adjacent normal tissue. Macrophages, mainly the M2 subtype, are dominant in TNBC tumors, accounting for 39.4% of all leukocytes (Figure S3B).

**FIGURE 2:**
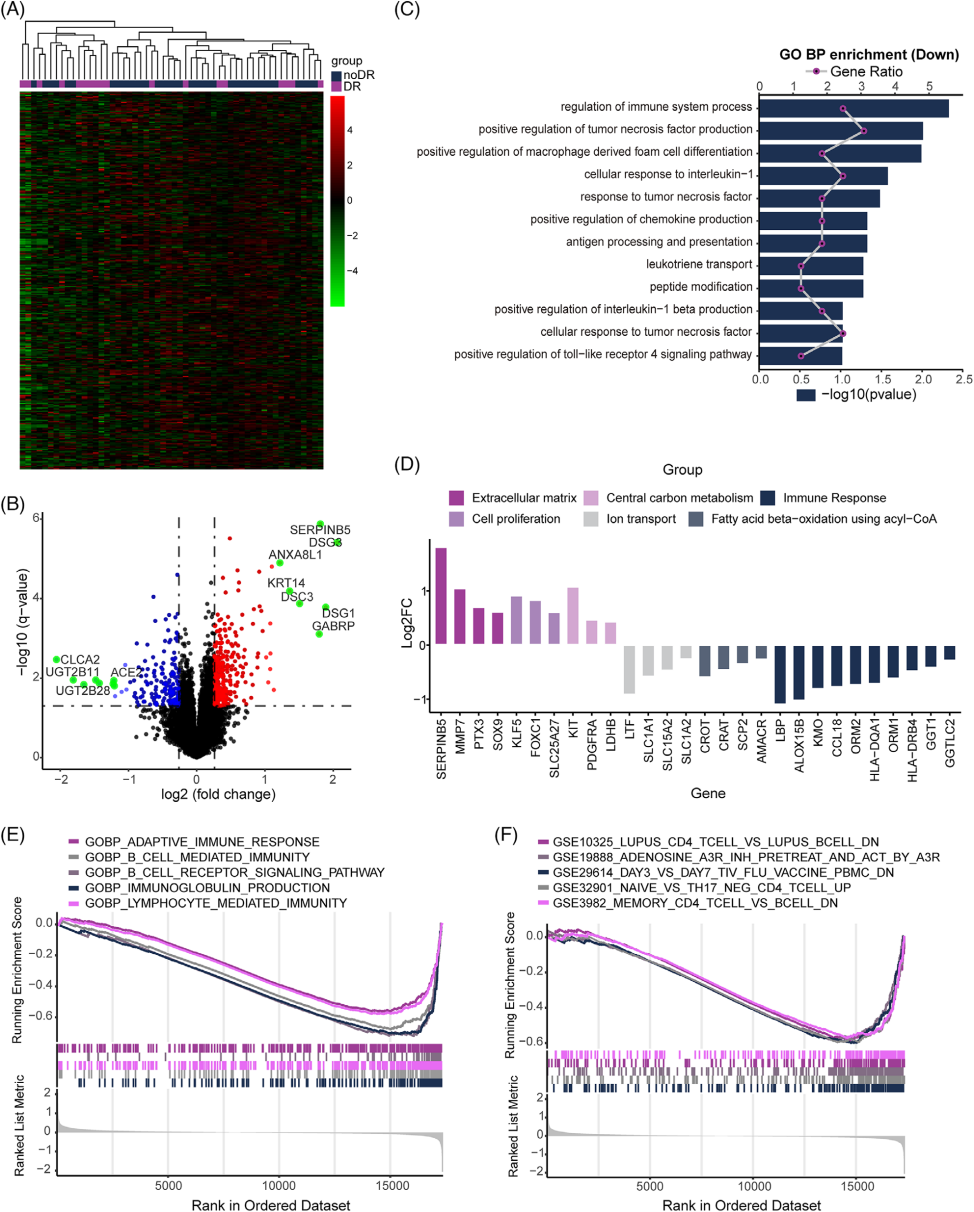
Transcriptome profiles of triple‐negative breast cancer (TNBC) patients with recurrence after adjuvant radiotherapy. (A) Hierarchical clustering of all differentially expressed genes (DEGs) in RT‐treated TNBC patients with recurrence and those without recurrence. Expression values are Z score transformed and are clustered by using Euclidean distance. (B) Volcano plot displaying 286 upregulated (red) and 172 downregulated (green) messenger RNAs (mRNAs) in RT‐treated TNBC patients with recurrence compared to those without recurrence. (C) Gene Ontology (GO) analysis revealing immune‐related biological processes enriched by downregulated genes in RT‐treated TNBC patients. (D) Bar plot of selected DEGs in TNBC patients with recurrence (*p *< .05). (E, F) Gene set enrichment analysis (GSEA) identifying top five enriched gene sets associated with immune responses in recurrent cases, annotated by C5_GO_BP and C7 collection (|NES| > 1, NOM *p*‐value < .05 and FDR q‐value < .25).

To explore the predictive value of TNBC molecular subtypes for tumor recurrence after RT, patients were categorized into four subtypes based on the FUSCC classification.^1^, ^6^ Notable disparities in recurrence‐free survival (RFS) across these TNBC subtypes were found (Figure 3A, *p *= .016). The BLIS subtype was associated with poorest RFS, evident in both univariate (*p *= .011, Figure 3B) and multivariate analyses (hazard ratio [HR]: 3.861; 95% confidence interval [CI]: 1.108–13.447; *p *= .034; Table S2). BLIS patients also exhibited significantly lower 12‐CK, co‐stimulatory molecules, CYT, and HLA molecules scores than other subtypes (Figure 3C), indicating suppressed immune activation. Combining RSI and FUSCC‐TNBC subtypes further refined recurrence risk assessment, with BLIS‐RR patients having markedly higher recurrence risks than other combined subtypes (Figures 3D,F). Multivariate analysis confirmed BLIS‐RR as the most significant RFS predictor, surpassing traditional clinicopathologic factors (Table S3; HR: 8.175; 95% CI: 1.939–34.454; *p *= .004).

**FIGURE 3:**
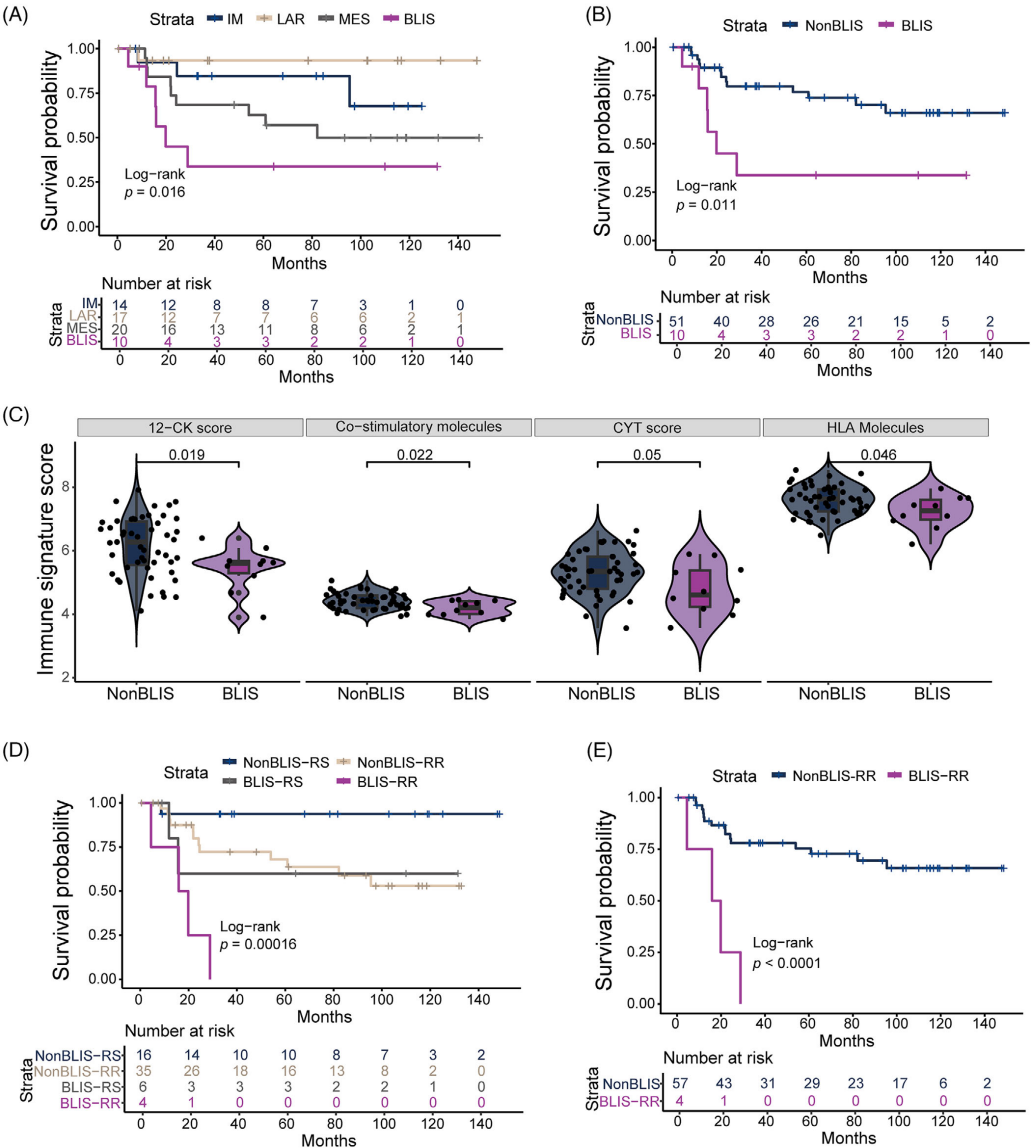
Integrating radiosensitivity index (RSI) signature with Fudan University Shanghai Cancer Center (FUSCC)‐triple‐negative breast cancer (TNBC) molecular subtypes refines the genomic classification of TNBC for recurrence‐free survival (RFS). (A) Kaplan‐Meier plot and log‐rank test comparing RFS among four subtypes according to the FUSCC classification. (B) Comparison of RFS between the basal‐like and immune‐suppressed (BLIS) subtype and other FUSCC subtypes. (C) Box plot illustrating differences in 12‐chemokine (12‐CK), co‐stimulatory molecules, cytolytic activity (CYT), and HLA molecules scores between the BLIS subtype and other subtypes. (D) Kaplan‐Meier plot and log‐rank test for RFS among four combined subtypes: BLIS‐RR, BLIS‐RS, nonBLIS‐RR and nonBLIS‐RS. (E) Analysis of RFS in the BLIS‐RR subtype compared to the combined other subtypes.

Among various differentially expressed genes, SERPINB5 was the most significantly upregulated in recurrent patients (Figure 2B,D and Figure S2) and notably higher in the BLIS‐RR subtype (Figure 4A). Existing literature indicates that SERPINB5 is associated with radioresistance and poor prognosis, highlighting its potential as a critical modulator of radiosensitivity in TNBC.^7^, ^8^ In our cohort, multivariate Cox regression analysis revealed that high SERPINB5 expression is associated with poor RFS (HR: 8.74; 95% CI: 1.97–38.88; *p *= .004; Table S4). We validated SERPINB5's high expression in TNBC using both the METABRIC and TCGA datasets (Figure 4B,C). In the METABRIC dataset, high SERPINB5 expression correlated with significantly poorer prognosis compared to low expression (Figure 4D). Similarly, in the TCGA dataset, high SERPINB5 expression was linked to poorer overall survival (HR: 8.91; 95% CI: 1.211–65.538; *p *= .032; Table S5). SERPINB5 expression positively correlated with tumor proliferation (Figure 4E and Figure S5C) and negatively with evaluated immune scores (Figure 4F,G and Figure S5A,B,D), suggesting a role in radioresistance. Knockdown experiments in TNBC cell lines demonstrated that siSERPINB5 cells showed reduced radioresistance compared to control cells (Figures 4H,I and Table S6) and alterations in DNA‐PKcs phosphorylation and γ‐H2AX expression (Figures S6A–C). Immunofluorescence detection indicated more γ‐H2AX foci in siSERPINB5 cells, suggesting increased DNA damage (Figure S6D–F).

**FIGURE 4:**
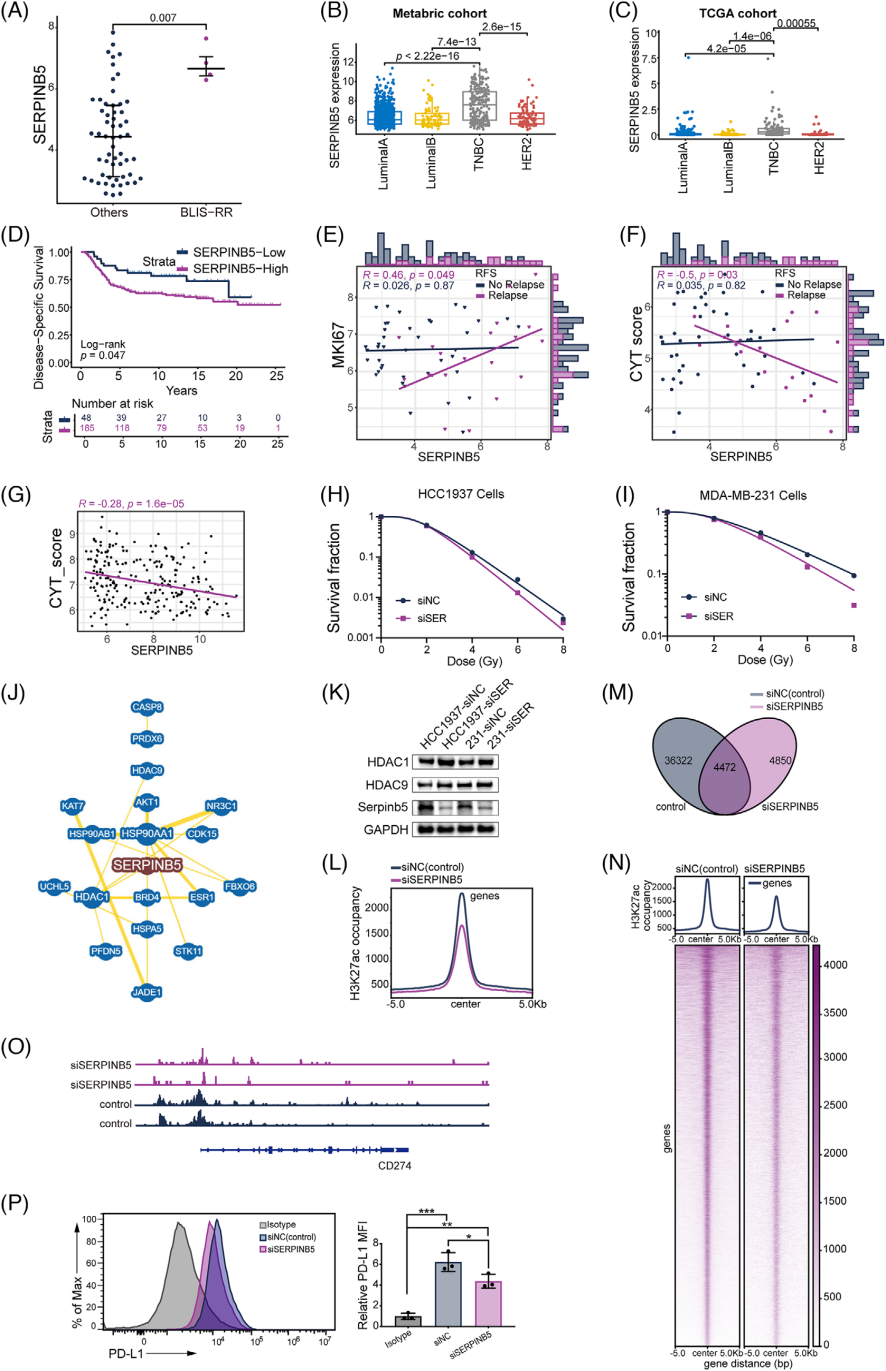
Exploring SERPINB5 as a key factor in triple‐negative breast cancer (TNBC) radioresistance. (A) Elevated SERPINB5 gene expression in the basal‐like and immune‐suppressed radioresistant (BLIS‐RR) subtype compared to other TNBC subtypes. (B, C) Box plots showing SERPINB5 expression across different breast cancer IHC subtypes in (B) the Metabric cohort and (C) the TCGA cohort, indicating significant overexpression in the TNBC subtype. (D) Kaplan‐Meier plot demonstrating disease‐specific survival differences between SERPINB5‐High and SERPINB5‐Low patients, highlighting poorer survival in the SERPINB5‐High group. (E) Dot plot showing the correlation between SERPINB5 and MKi67 expression in TNBC patients categorized by different recurrence‐free survival (RFS) outcomes. (F) Dot plot revealing the correlation between SERPINB5 expression and cytolytic activity (CYT) score in TNBC patients with different RFS. (G) Dot plot revealing the negative correlation between SERPINB5 expression and the CYT score in the Metabric TNBC cohort. (H, I) Clonogenic survival analyses measuring the radiosensitizing effects of knocking down the SERPINB5 gene in (H) HCC1937 and (I) MDA‐MB‐231 cells. (J) Computational analysis revealing the protein interaction network associated with SERPINB5. (K) Western blot analysis assessing the impact of SERPINB5 knockdown on HDAC1 and HDAC9 expression levels in HCC1937 and MDA‐MB‐231 cells. (L) CUT&Tag metagene profiling contrasting SERPINB5 gene knockdown with the control condition. (M) Peak distribution profile in the control and following SERPINB5 gene knockdown. (N) Heatmap illustrating the genomic landscape changes induced by SERPINB5 gene knockdown compared to the control. (O) Representative track examples showing reduced H3K27ac levels in DNA and CD274 following SERPINB5 knockdown. (P) Flow cytometry analysis demonstrating the effect of SERPINB5 knockdown on the surface expression of PD‐L1 in MDA‐MB‐231 cells.

To further understand the mechanisms underlying the impact of SERPINB5 on antitumor immunity and radioresistance, we conducted preliminary explorations, providing a direction for further investigation. Previous studies identified SERPINB5 as an endogenous inhibitor of HDACs.^9^, ^10^ Bioinformatics analysis suggested a close association between SERPINB5 and HDACs (Figure 4J). We experimentally validated this interaction, showing that SERPINB5 knockdown leads to increased expression of HDAC1 and HDAC9 by western blot analysis (Figure 4K). SERPINB5 knockdown reduced chromatin H3K27ac levels, altering the chromatin status (Figures 4L–N). Additionally, the knockdown impacted the epigenetic regulation of CD274 (encoding PD‐L1), linking SERPINB5 to immune modulation and radioresistance (Figure 4O). Flow cytometry analysis confirmed a reduction in PD‐L1 surface expression on SERPINB5 knockdown MDA‐MB‐231cell lines (Figure 4P). However, in‐depth experimental research is warranted to fully elucidate the molecular mechanisms of the interaction between SERPINB5 and HDACs, as well as its specific roles in RT and immunotherapy.

In conclusion, our research highlights the BLIS‐RR subtype, identified by integrating RSI with FUSCC TNBC classification, as particularly susceptible to radioresistance and recurrence post‐RT. Crucially, SERPINB5 is identified as a key factor linked to radioresistance in this subtype, potentially through its interaction with HDACs and impact on chromatin structure and immune modulation. These findings provide valuable insights for personalized RT approaches in high‐risk TNBC.

## AUTHOR CONTRIBUTIONS

Xinxin Rao, Xuanyi Wang, Chao He and YiZhou Jiang performed the investigation and formal analysis. Xinxin Rao and Xuanyi Wang also contributed to writing the original draft. ZhiMing Shao provided resources and performed the investigation. Yan Feng was responsible for investigation and supervision. Jundong Zhou and Xingxing Chen conceptualized the project, provided resources and contributed to writing and editing the manuscript. Xiaomao Guo and Xingxing Chen were involved in funding acquisition, supervision and writing—review and editing.

## CONFLICT OF INTEREST STATEMENT

The authors declare no conflict of interest.

## FUNDING INFORMATION

This study was supported by the National Natural Science Foundation of China (81602668), the Innovation Program of Shanghai Municipal Education Commission (21140900900) and the Key Clinical Specialty Project of Shanghai.

## ETHICS STATEMENT

All tissue samples were obtained with the approval of the independent ethical committee/institutional review board at Fudan University Shanghai Cancer Center Ethical Committee.

## PATIENT CONSENT STATEMENT

Each patient signed an informed consent form.

## Supporting information

Supporting Information

Supporting Information

Supporting Information

## Data Availability

Datasets are available upon request. Contact Xingxing Chen (xingxingfdu@hotmail.com).
