# Supplementary material for: Integrating radiosensitivity index and triple‐negative breast cancer subtypes reveals SERPINB5 as a radioresistance biomarker in triple‐negative breast cancer
Source: Clin Transl Med. 2024 Aug 7;14(8):e1787. doi: 10.1002/ctm2.1787 (PMC11306282; doi:10.1002/ctm2.1787)
Supplement: Supplementary file 1 — Supporting Information [file CTM2-14-e1787-s002.docx]

**Materials and Methods**

***Patient recruitment***

From 1 January 2011 to 31 December 2012, 160 consecutive patients diagnosed with TNBC who were treated in our center and willing to participate in our study were included as previously described^1^. Recurrence-free survival (RFS) was defined as the time from the date of surgery to the date of confirmed disease recurrence or the date of the last follow-up visit for recurrence-free patients.

***Transcriptome microarray and functional annotation***

Total RNA was extracted from 160 frozen TNBC samples and 9 paired adjacent normal breast tissues. The Affymetrix Human Transcriptome Array 2.0 GeneChips (Affymetrix, CA, USA) were employed for transcriptome profiling. Raw gene expression data underwent normalization using the Robust Multi-array Average (RMA) method. Differential gene expression analysis was conducted between patients with and without recurrence, utilizing the "limma" package in Bioconductor and R. Gene Ontology (GO) analysis and Gene Set Enrichment Analysis (GSEA) were performed to explore the biological functions of differentially expressed genes (DEGs)^2-4^. Immune cytolytic activity (CYT) was quantified as the geometric mean transcript levels of two key cytolytic effectors, granzyme A (*GZMA*) and perforin (*PRF1*)^5^. Twelve-chemokine (12-CK) score represented the median transcript value of 12 chemokine genes^6^ (CCL2, CCL3, CCL4, CCL5, CCL8, CCL18, CCL19, CCL21, CXCL9, CXCL10, CXCL11 and CXCL13). Co−stimulatory molecules score and HLA molecules score were defined as the mean transcript levels of 19 immune-related gene (CD28, CD40, CD40LG, CD70, CD86, ICOS, ICOSLG, IL6, IL6R, TNFSF4, TNFRSF4, TNFRSF9, TNFSF13, TNFSF13B, TNFRSF13B, TNFRSF17, TNFRSF18, TNFSF18, TMEM173) and 20 HLA related molecules (B2M, HLA-A, HLA-B, HLA-C, TAP1, TAP2, HLA-DMA, HLA-DMB, HLA-DOA, HLA-DOB, HLA-DPA1, HLA-DPB1, HLA-DPB2, HLA-DQA1, HLA-DQA2, HLA-DQB1, HLA-DQB2, HLA-DRA, HLA-DRB1, HLA-DRB5), respectively^7^. The immune signature (IMS) score was calculated as follows:

IMS_score=4.7*ADRM1+3.6*MICB+4.8*PSMD13-3.7*RFXANK

***Assessment of immune infiltration by the CIBERSORT algorithm***

The CIBERSORT deconvolution algorithm was applied to estimate the relative fractions of 22 immune cell types in tumor tissue or corresponding normal para-tumor tissue, as described previously^8^. The LM22 signature was calculated using 1000 permutations, with other parameters set to default values.

***Radiosensitivity molecular signature index (RSI) determination***

The RSI, initially developed in 48 cancer cell lines to predict cellular radiosensitivity based on the survival fraction at 2 Gy^9^, has been validated in multiple cancer cohorts^6,10^. For this study, the 35th percentile of RSI served as the cutoff point. RSI-low patients (more radiosensitive, RS) were defined as having an RSI ≤ 0.765, and RSI-high patients (more radioresistant, RR) were defined as having an RSI>0.765.

***Real-time quantitative reverse transcription-PCR (qRT–PCR)***

Total RNA from patient samples was extracted using TRIzol reagent (Invitrogen, Carlsbad, CA). cDNAs were synthesized using the ExScript RT–PCR kit (Takara Bio Inc., Japan). Eight recurrence-related mRNAs were selected for qRT–PCR with primers listed in Table S7. qPCRs were conducted on a LightCycler 480 system (Roche, Basel, Switzerland) using LightCycler 480 SYBR Green I Master Mix (Roche, Basel, Switzerland), in triplicate. Relative quantification of target genes utilized the ΔΔCt method with β-actin as the internal control.

***Knockdown or overexpression of SERPINB5***

Small double-strand interfering RNAs (siRNAs) targeting *SERPINB5* mRNA or negative control mRNA sequences (Table S8) were transfected into HCC1937 and MDA-MB-231 cells to achieve *SERPINB5* knockdown. Lentiviral vectors containing the *SERPINB5* gene sequence or negative control were transfected into cells for *SERPINB5* overexpression. GFP expression served as a marker for lentiviral transfection detection.

***Cell cultures, Immunofluorescence and Western blot analysis,***

The human TNBC cell line HCC1937 (ATCC^®^ CRL-2336) and MDA-MB-231 (ATCC^®^ HTB-26^TM^) was obtained from ATCC. Cell cultures, Immunofluorescence and Western blot analysis were performed as previously described^11,12^.

***Radiation source and Clonogenic survival assay***

Exponentially growing TNBC cells were harvested, counted, and seeded at 500 cells per well in 6-well plates. After overnight incubation for attachment, cells were irradiated at 0, 2, 4, 6 and 8 Gy using an X-RAD 320 Biological Irradiator (Precision X-ray Inc) at a dose rate of 246 cGy/min (50 cm from the radiation source [SSD] with 250 kVp x-rays, using 12 mA and a filter consisting of 2.5 mm Al and 0.1 mm Cu). All cells were then cultured for 12 days, followed by the removal of the culture medium. Cells were fixed with methanol for 20 minutes and stained with 0.25% crystal violet solution for 20 minutes. Colonies consisting of at least 50 cells were counted under a microscope. Plating efficiency (PE, %) was calculated for the 0 Gy treatment group as PE = (number of colonies / number of seeded cells) x 100%. The surviving fraction (SF, %) for each group was calculated as SF = (number of colonies / number of seeded cells) x PE. Cell survival curves were fitted using the single-hit multi-target model to determine radiobiological parameters, including D_0_, D_q_, SF_2_, and the sensitization enhancement ratio (SER).

***Cleavage Under Targets and Tagmentation (CUT&Tag)***

CUT&Tag was performed following the published protocol^13,14^ with minor modifications. Briefly, nuclei (100-150k) were isolated using NE1 buffer and bound to ConA magpoly beads (Smart-lifesciences, SM04102). Primary antibody targeting H3 acetylation at lysine 27 (H3K27ac) (Abclonal, A7253) was incubated with nuclei in primary antibody buffer for 2 h at room temperature. Subsequently, a secondary antibody (Goat anti-rabbit IgG, Novoprotein, N269) was incubated with the nuclei in dig-wash buffer for 1 h. Purified pAG-Tn5 transposase was then added in dig-300 buffer for tagmentation and target DNA cleavage. Following incubation with tagmentation buffer, the extracted DNA was amplified by PCR and purified using VAHTS DNA clean beads (VAHTS, N411). The pooled library was subjected to paired-end sequencing using HiSeqX (Illumina) with 150 bp read length.

***Data processing of CUT&Tag***

After quality control, reads were aligned against hg38 by Bowtie2 (version 2.3.5.1). Peak calling was performed by SEACR (version 1.4) for H3K27ac with relaxed mode. A heatmap was generated using deepTools (version 3.3.2, binSize = 10).

***Protein-protein interaction (PPI)***

Protein-protein interaction (PPI) analysis was performed using BioGrid and STRING with default settings.

***Statistical Analysis***

Statistical analysis was performed using GraphPad Prism 8 software, R software (version 3.6.2) and STATA (version 11.0). Mean value differences were tested by using the t test, ANOVA test, and Kruskal-Wallis test depending on the variance and number of compared groups. Tukey’s multiple comparisons and Benjamini-Hochberg procedures were used to correct multiple testing errors. Kaplan-Meier survival analyses were performed to compare clinical outcomes between subgroups. Multivariate analysis was performed using Cox logistic regression. The significance level was set at *p* < 0.05.

**References**

1. Liu, Y.R., Jiang, Y.Z., Xu, X.E., Yu, K.D., Jin, X., Hu, X., Zuo, W.J., Hao, S., Wu, J., Liu, G.Y., et al. (2016). Comprehensive transcriptome analysis identifies novel molecular subtypes and subtype-specific RNAs of triple-negative breast cancer. Breast Cancer Res *18*, 33. 10.1186/s13058-016-0690-8.

2. Huang da, W., Sherman, B.T., and Lempicki, R.A. (2009). Systematic and integrative analysis of large gene lists using DAVID bioinformatics resources. Nat Protoc *4*, 44-57. 10.1038/nprot.2008.211.

3. Huang da, W., Sherman, B.T., and Lempicki, R.A. (2009). Bioinformatics enrichment tools: paths toward the comprehensive functional analysis of large gene lists. Nucleic Acids Res *37*, 1-13. 10.1093/nar/gkn923.

4. Yu, G., Wang, L.G., Han, Y., and He, Q.Y. (2012). clusterProfiler: an R package for comparing biological themes among gene clusters. Omics : a journal of integrative biology *16*, 284-287. 10.1089/omi.2011.0118.

5. Rooney, M.S., Shukla, S.A., Wu, C.J., Getz, G., and Hacohen, N. (2015). Molecular and genetic properties of tumors associated with local immune cytolytic activity. Cell *160*, 48-61. 10.1016/j.cell.2014.12.033.

6. Strom, T., Harrison, L.B., Giuliano, A.R., Schell, M.J., Eschrich, S.A., Berglund, A., Fulp, W., Thapa, R., Coppola, D., Kim, S., et al. (2017). Tumour radiosensitivity is associated with immune activation in solid tumours. Eur J Cancer *84*, 304-314. 10.1016/j.ejca.2017.08.001.

7. Kraya, A.A., Maxwell, K.N., Wubbenhorst, B., Wenz, B.M., Pluta, J., Rech, A.J., Dorfman, L.M., Lunceford, N., Barrett, A., Mitra, N., et al. (2019). Genomic Signatures Predict the Immunogenicity of BRCA-Deficient Breast Cancer. Clin Cancer Res *25*, 4363-4374. 10.1158/1078-0432.Ccr-18-0468.

8. Newman, A.M., Liu, C.L., Green, M.R., Gentles, A.J., Feng, W., Xu, Y., Hoang, C.D., Diehn, M., and Alizadeh, A.A. (2015). Robust enumeration of cell subsets from tissue expression profiles. Nat Methods *12*, 453-457. 10.1038/nmeth.3337.

9. Eschrich, S., Zhang, H., Zhao, H., Boulware, D., Lee, J.H., Bloom, G., and Torres-Roca, J.F. (2009). Systems biology modeling of the radiation sensitivity network: a biomarker discovery platform. Int J Radiat Oncol Biol Phys *75*, 497-505. 10.1016/j.ijrobp.2009.05.056.

10. Eschrich, S.A., Fulp, W.J., Pawitan, Y., Foekens, J.A., Smid, M., Martens, J.W., Echevarria, M., Kamath, V., Lee, J.H., Harris, E.E., et al. (2012). Validation of a radiosensitivity molecular signature in breast cancer. Clinical cancer research : an official journal of the American Association for Cancer Research *18*, 5134-5143. 10.1158/1078-0432.CCR-12-0891.

11. Zhou, Z.R., Yang, Z.Z., Wang, S.J., Zhang, L., Luo, J.R., Feng, Y., Yu, X.L., Chen, X.X., and Guo, X.M. (2017). The Chk1 inhibitor MK-8776 increases the radiosensitivity of human triple-negative breast cancer by inhibiting autophagy. Acta pharmacologica Sinica *38*, 513-523. 10.1038/aps.2016.136.

12. Chen, X., Ma, N., Zhou, Z., Wang, Z., Hu, Q., Luo, J., Mei, X., Yang, Z., Zhang, L., Wang, X., et al. (2017). Estrogen Receptor Mediates the Radiosensitivity of Triple-Negative Breast Cancer Cells. Medical science monitor : international medical journal of experimental and clinical research *23*, 2674-2683. 10.12659/msm.904810.

13. Kaya-Okur, H.S., Wu, S.J., Codomo, C.A., Pledger, E.S., Bryson, T.D., Henikoff, J.G., Ahmad, K., and Henikoff, S. (2019). CUT&Tag for efficient epigenomic profiling of small samples and single cells. Nat Commun *10*, 1930. 10.1038/s41467-019-09982-5.

14. Kaya-Okur, H.S., Janssens, D.H., Henikoff, J.G., Ahmad, K., and Henikoff, S. (2020). Efficient low-cost chromatin profiling with CUT&Tag. Nat Protoc *15*, 3264-3283. 10.1038/s41596-020-0373-x.
