# Supplementary material for: Integrating radiosensitivity index and triple‐negative breast cancer subtypes reveals SERPINB5 as a radioresistance biomarker in triple‐negative breast cancer
Source: Clin Transl Med. 2024 Aug 7;14(8):e1787. doi: 10.1002/ctm2.1787 (PMC11306282; doi:10.1002/ctm2.1787)

**Supplementary Figures and legends**

**
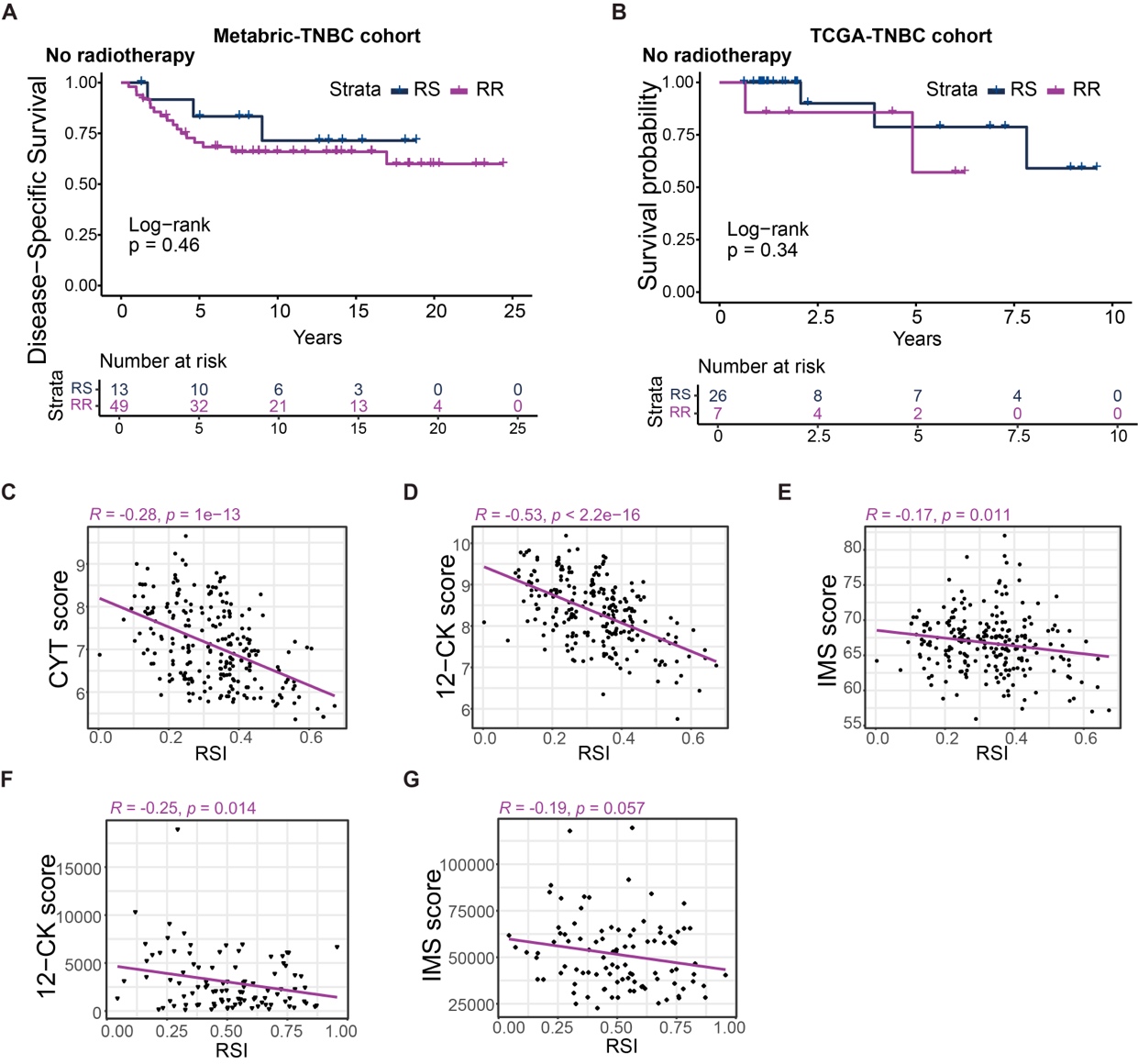
**

**Figure S1**. Association of the radiosensitivity index (RSI) signature with survival outcomes and immune repression in public datasets.

(A) Kaplan-Meier plot showing RFS comparison between radiosensitive (RS) and radioresistant (RR) patients without radiotherapy (RT) in the Metabric triple-negative breast cancer (TNBC) cohort. (B) Kaplan-Meier plot showing RFS comparison between RS and RR patients without RT in the TCGA TNBC cohort. (C-E) Dot plots demonstrating the correlation between RSI and scores of (C) cytolytic activity (CYT), (D) 12-chemokine (12-CK), and (E) immune signature (IMS) in the Metabric TNBC cohort. (F, G) Dot plots demonstrating the correlation between RSI and scores of (F) 12-CK and (G) IMS in the TCGA TNBC cohort.

**
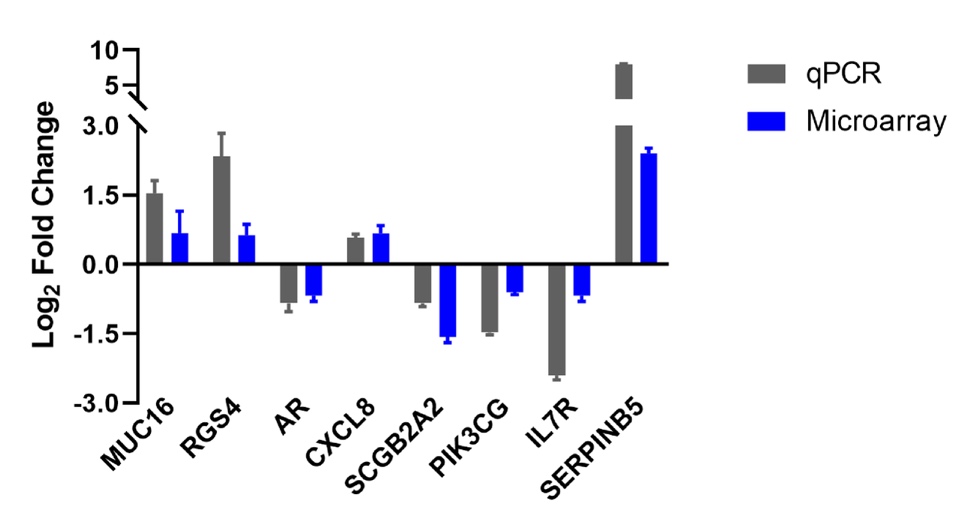
**

**Figure S2**. qRT-PCR validation of differentially expressed genes (MUC16, RGS4, CXCL8, AR, SERPINB5, SCGB2A2, PIK3CG, and CCL19) identified in microarray data.


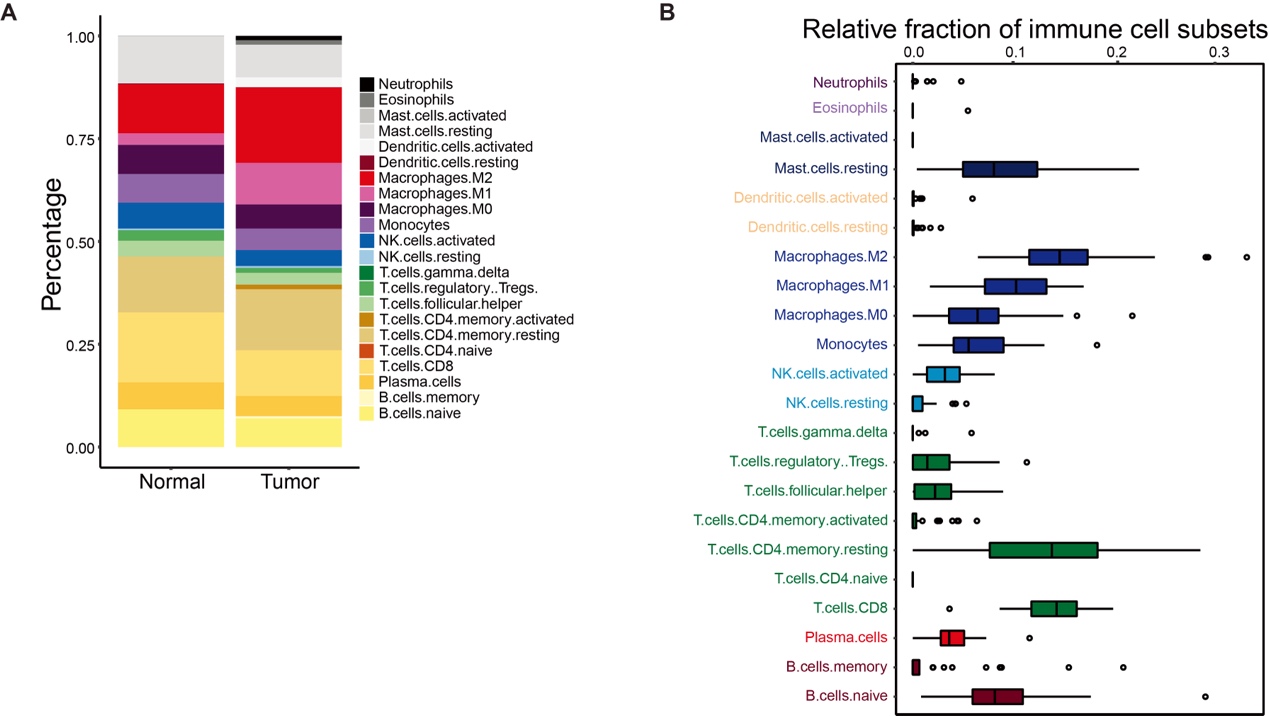


**Figure S3**. The landscape of immune cell infiltration in TNBC patients.

(A) The difference in immune infiltration between adjacent tissues (normal) and tumor tissues (tumor). (B) Distribution of 22 immune cells subsets in TNBC tumor tissue.


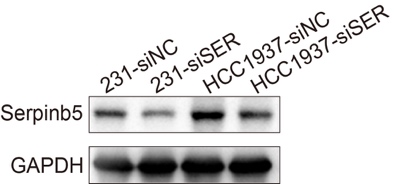


**Figure S4**. Western blot analysis depicting the protein levels of Serpinb5 in MDA-MB-231 and HCC1937 cells after knockdown.


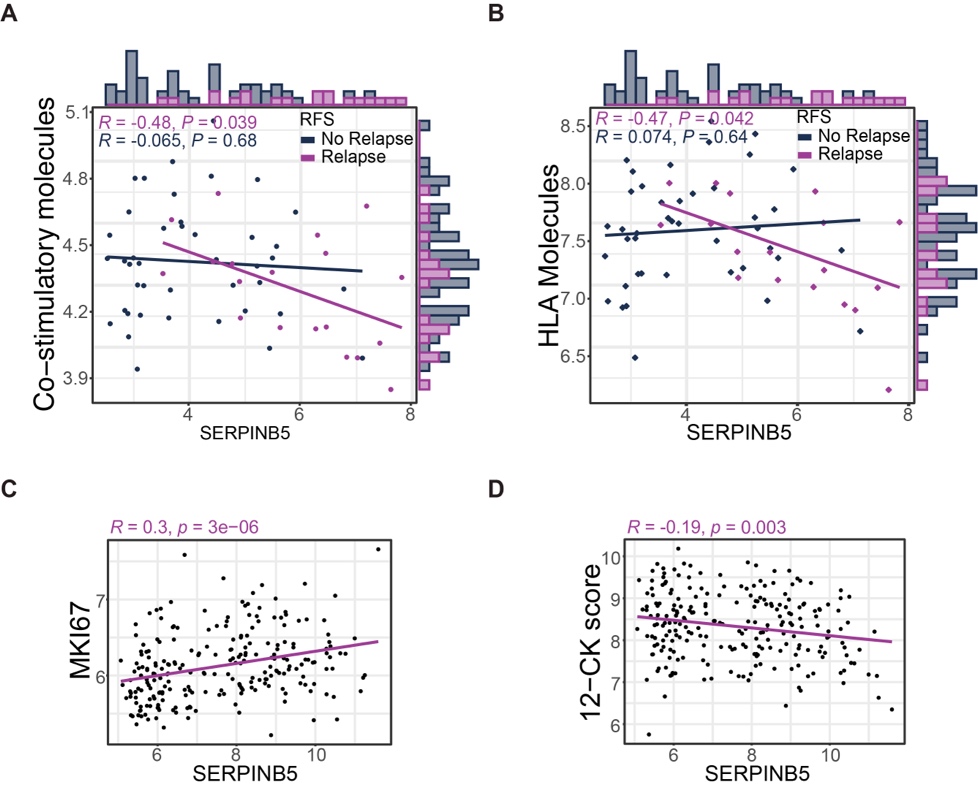


**Figure S5**. Correlation between SERPINB5 expression and immune-related scores in TNBC patients.

(A) Dot plot revealing the correlation between SERPINB5 expression and the co-stimulatory molecules score in FUSCC TNBC patients with different RFS. (B) Dot plot revealing the correlation between SERPINB5 expression and the HLA molecules score in FUSCC TNBC patients with different RFS. (C) Dot plot showing the positive correlation between SERPINB5 and MKi67 expression in the Metabric TNBC cohort. (D) Dot plot revealing the negative correlation between SERPINB5 expression and the 12-chemokine (12-CK) score in the Metabric TNBC cohort.


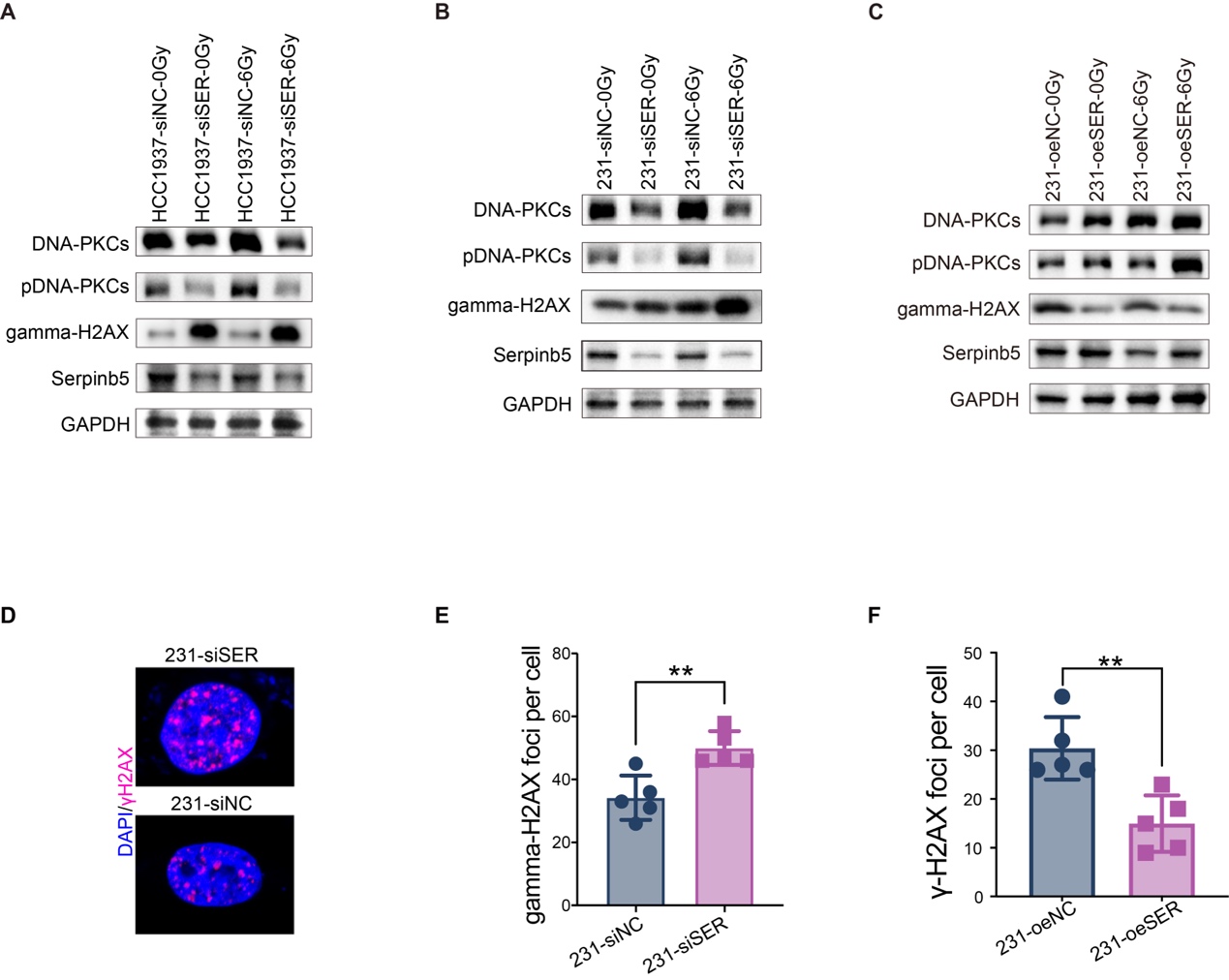


**Figure S6**. Evaluating the effects of SERPINB5 modulation on DNA damage response in TNBC cell lines.

(A, B) Western blot analysis assessing the impact of SERPINB5 knockdown on DNA-PKs, phosphorylated DNA-PKs (p-DNA-PKs), and γ-H2AX expression levels in (A) HCC1937 and (B) MDA-MB-231 cells. (C) Western blot analysis examining the effects of SERPINB5 overexpression on DNA-PKs, p-DNA-PKs, and γ-H2AX expression in MDA-MB-231 cells. (D) Representative images of γH2AX staining in MDA-MB-231 cells with SERPINB5 knockdown compared to control cells. (E, F) Quantification of γH2AX foci in MDA-MB-231 cells following SERPINB5 (E) knockdown and (F) overexpression, relative to controls.

**Original western blots from Figure S4**


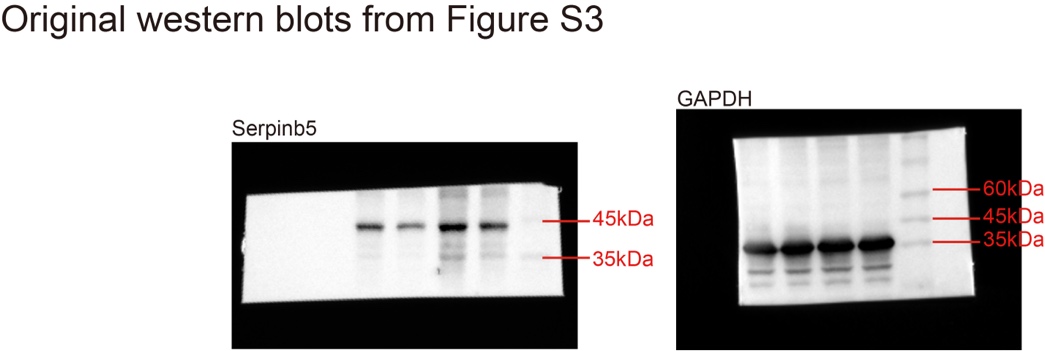


**Original western blots from Figure 4K**


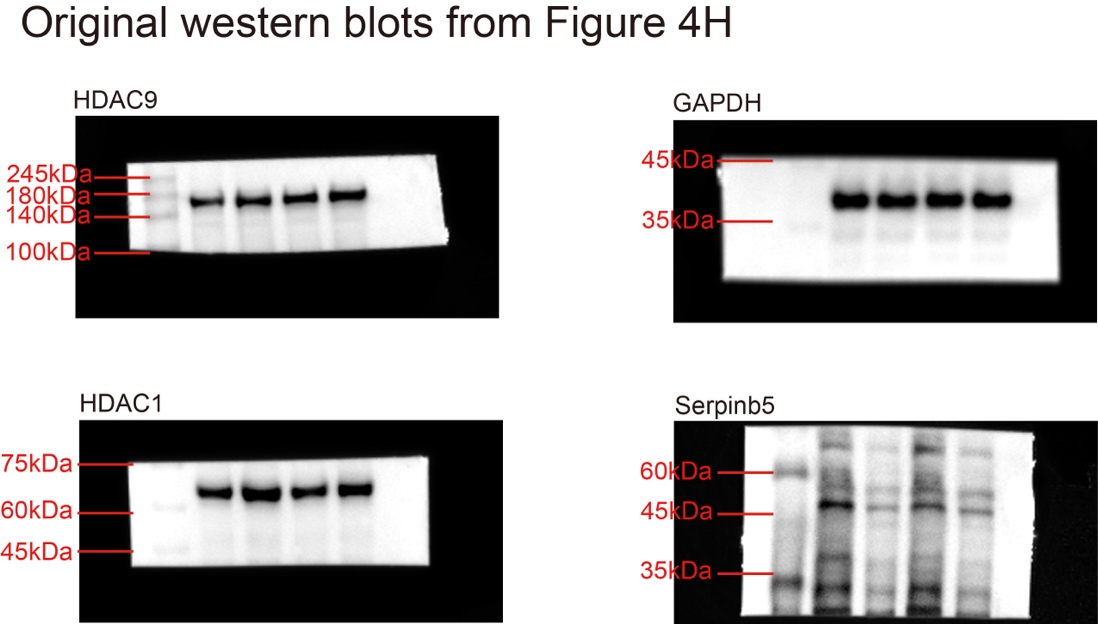


**Original western blots from Figure S6A**


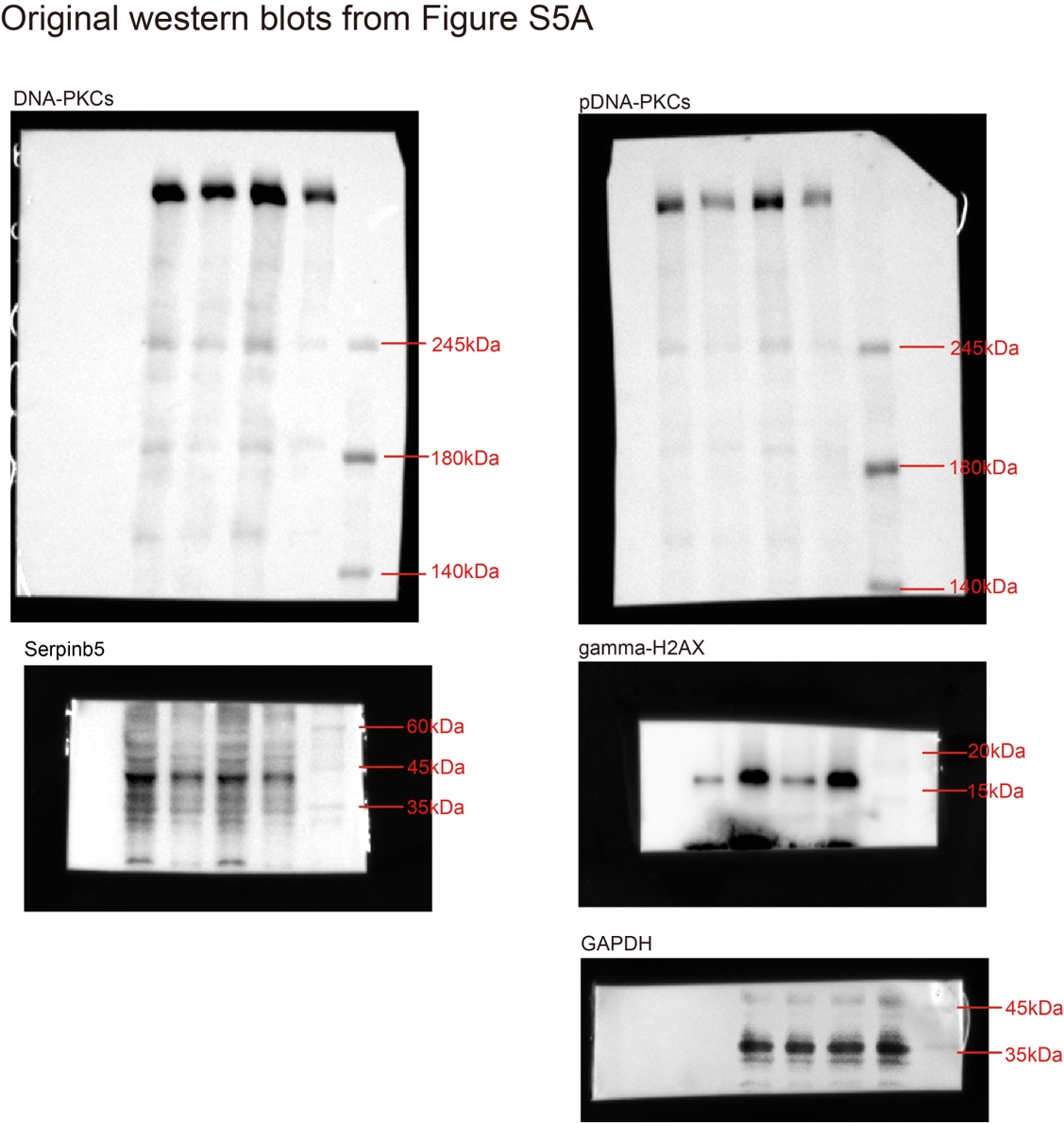


**Original western blots from Figure S6B**


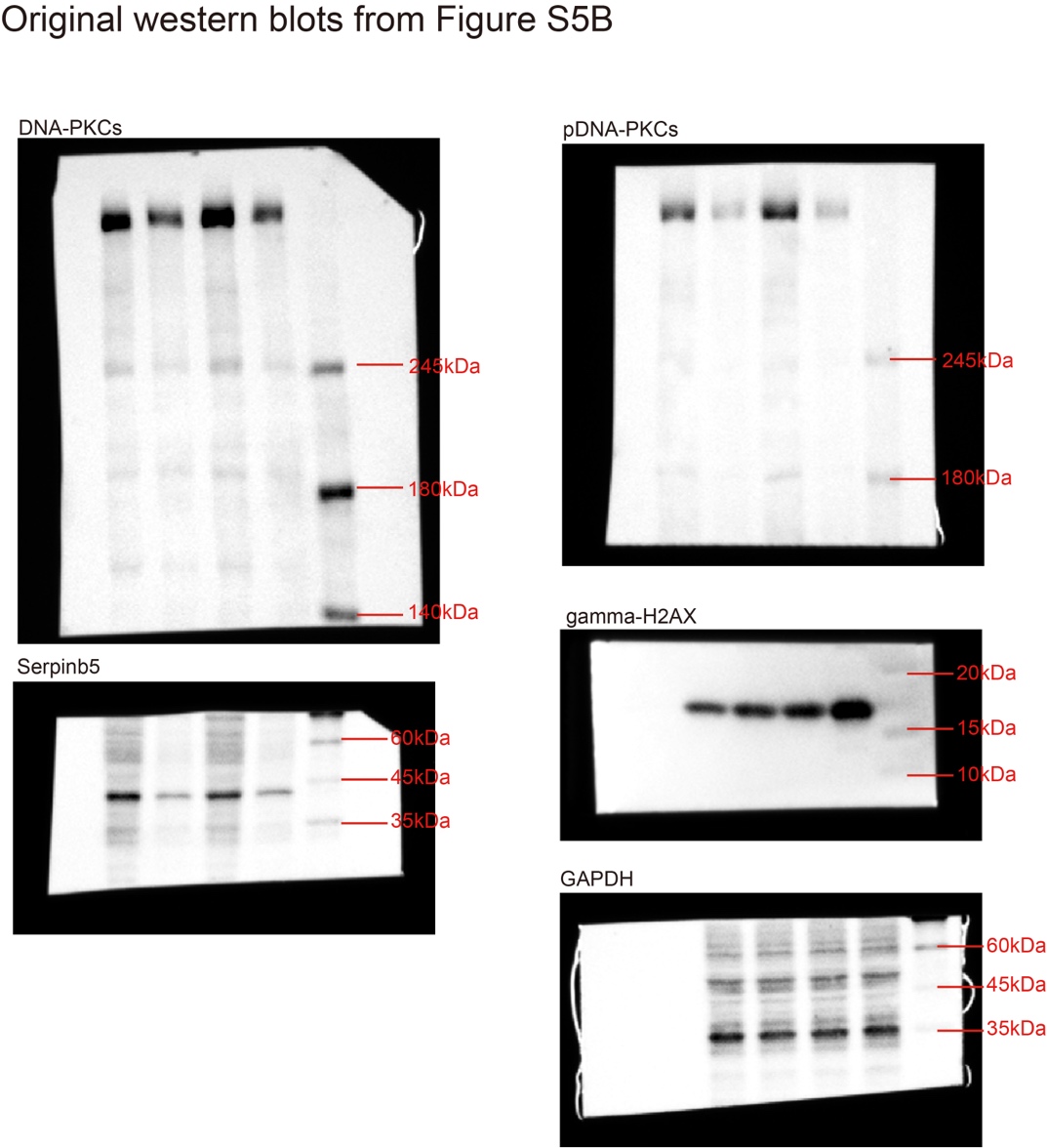


**Original western blots from Figure S6C**


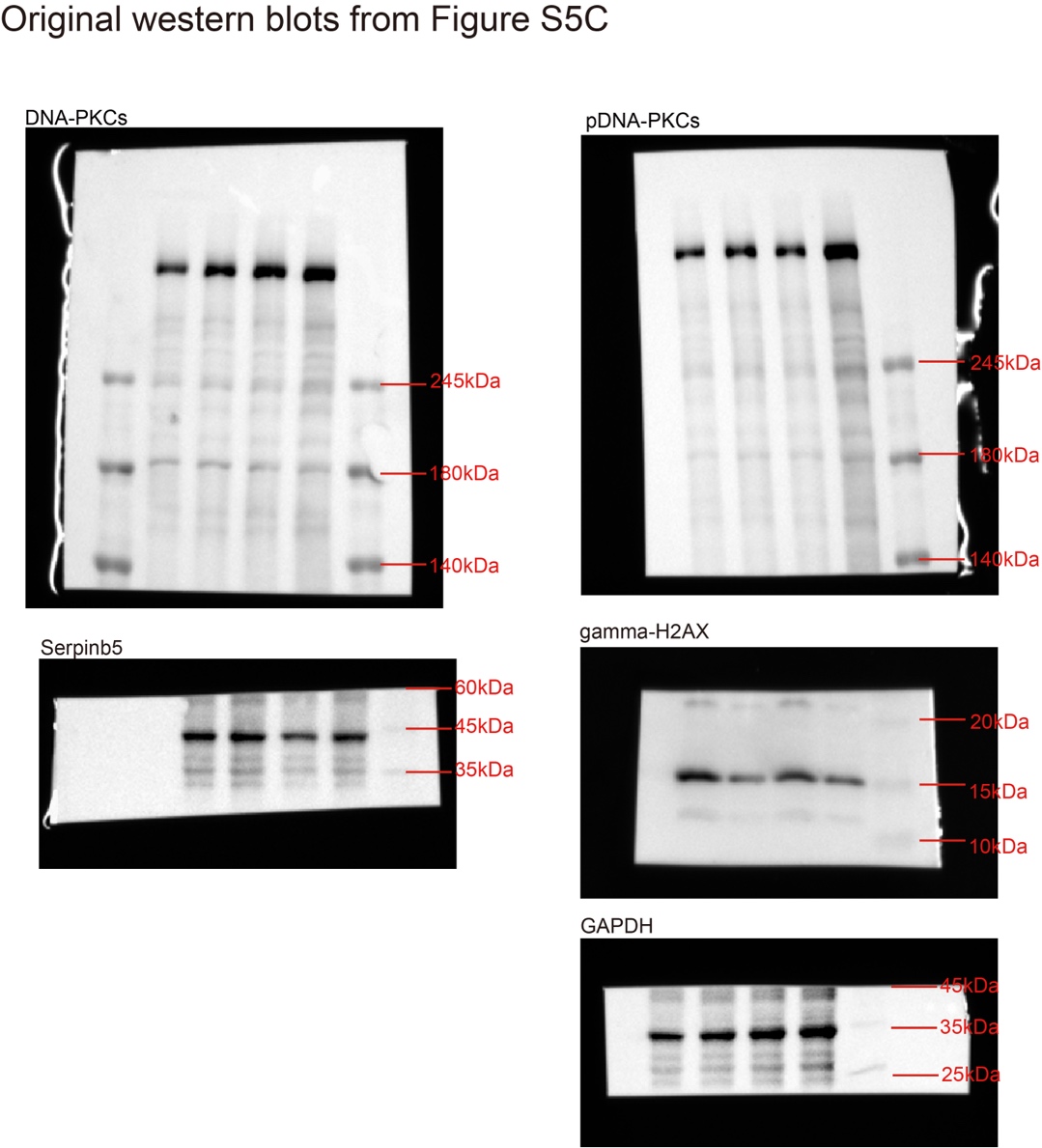

Supplement: Supplementary file 2 — Supporting Information [file CTM2-14-e1787-s003.docx]
