# Supplementary material for: Integrating radiosensitivity index and triple‐negative breast cancer subtypes reveals SERPINB5 as a radioresistance biomarker in triple‐negative breast cancer
Source: Clin Transl Med. 2024 Aug 7;14(8):e1787. doi: 10.1002/ctm2.1787 (PMC11306282; doi:10.1002/ctm2.1787)
Supplement: Supplementary file 3 — Supporting Information [file CTM2-14-e1787-s001.docx]

**Table S1. Clinicopathologic characteristics of 160 patients with TNBC**

| **Characteristics** | **Total (%)**  **(N=160)** | **RS**  **(N=56)** | **RR**  **(N=104)** | ***P value*** |
| --- | --- | --- | --- | --- |
| **Age (years)** |  |  |  |  |
| **<50** | 54 (33.8%) | 20 (35.7%) | 34 (32.7%) | 0.833 |
| **≥50** | 106 (66.3%) | 36 (64.3%) | 70 (67.3%) |  |
| **Menopausal status** |  |  |  |  |
| **Yes** | 98 (61.3%) | 34 (60.7%) | 64 (61.5%) | 1 |
| **No** | 62 (38.8%) | 22 (39.3%) | 40 (38.5%) |  |
| **Tumor size (cm)** |  |  |  |  |
| **≤2** | 57 (35.6%) | 20 (35.7%) | 37 (35.6%) | 0.903 |
| **>2 and ≤5** | 101 (63.1%) | 35 (62.5%) | 66 (63.5%) |  |
| **>5** | 2 (1.3%) | 1 (1.8%) | 1 (1.0%) |  |
| **Grade** |  |  |  |  |
| **I-II** | 50 (31.3%) | 13 (23.2%) | 37 (35.6%) | 0.093 |
| **III** | 87 (54.4%) | 37 (66.1%) | 50 (48.1%) |  |
| **Unknown** | 23 (14.4%) | 6 (10.7%) | 17 (16.3%) |  |
| **LVI** |  |  |  |  |
| **Yes** | 92 (57.5%) | 33 (58.9%) | 59 (56.7%) | 0.92 |
| **No** | 68 (42.5%) | 23 (41.1%) | 45 (43.3%) |  |
| **Excised LNs** |  |  |  |  |
| **1-10** | 38 (23.8%) | 12 (21.4%) | 26 (25.0%) | 0.771 |
| **11-20** | 73 (45.6%) | 25 (44.6%) | 48 (46.2%) |  |
| **>20** | 49 (30.6%) | 19 (33.9%) | 30 (28.8%) |  |
| **Positive LN_S_** |  |  |  |  |
| **0** | 88 (55.0%) | 31 (55.4%) | 57 (54.8%) | 0.945 |
| **1-3** | 40 (25.0%) | 14 (25.0%) | 26 (25.0%) |  |
| **4-9** | 14 (8.8%) | 4 (7.1%) | 10 (9.6%) |  |
| **≥10** | 18 (11.3%) | 7 (12.5%) | 11 (10.6%) |  |
| **Ki67 (%)** |  |  |  |  |
| **≤20** | 27 (16.9%) | 3 (5.4%) | 24 (23.1%) | 0.008 |
| **>20** | 133 (83.1%) | 53 (94.6%) | 80 (76.9%) |  |
| **Surgery** |  |  |  |  |
| **Mastectomy+ALND** | 118(73.8%) | 43(76.8%) | 75(72.1%) | 0.808 |
| **Mastectomy+SLNB** | 41(25.6%) | 13(23.2%) | 28(26.9%) |  |
| **Breast-conserving** | 1(0.6%) | 0(0%) | 1(1.0%) |  |
| **Chemotherapy** |  |  |  |  |
| **Taxane based** | 123 (76.9%) | 41 (73.2%) | 82 (78.8%) | 0.363 |
| **Anthracyline based** | 19 (11.9%) | 6 (10.7%) | 13 (12.5%) |  |
| **Others** | 18 (11.3%) | 9 (16.1%) | 9 (8.7%) |  |
| **Radiotherapy** |  |  |  |  |
| **Yes** | 61 (38.1%) | 24 (42.9%) | 37 (35.6%) | 0.463 |
| **No** | 99 (61.9%) | 32 (57.1%) | 67 (64.4%) |  |
| **RFS events** |  |  |  |  |
| **Yes** | 36 (22.5%) | 10 (17.9%) | 26 (25.0%) | 0.405 |
| **No** | 124 (77.5%) | 46 (82.1%) | 78 (75.0%) |  |

Abbreviations: LVI, lymphovascular invasion; LN, lymph node; TNBC, triple-negative breast cancer; RSI, radiosensitivity index; RR, radioresistance subtype; RS, radiosensitive subtype; RFS, recurrence-free survival.

**Table S2. Multivariate analysis modeling with RSI, BLIS subtype and clinicopathologic variables for RFS in TNBC patients treated with RT**

| **Variable^a^** | **RFS** | |
| --- | --- | --- |
|  | **HR (95% CI)** | ***P*** |
| **RSI-subtype, RR vs. RS** | 5.410 (1.451-20.169) | 0.012 |
| **FUSCC-subtype, BLIS vs. others** | 3.861 (1.108-13.447) | 0.034 |
| **Age (y), ≤40 vs. >40** | 3.716 (1.072-12.880) | 0.038 |
| **Tumor size, ≥3.5cm vs. <3.5cm** | 0.307 (0.036-2.557) | 0.275 |
| **Lymph nodes, >4 vs. ≤3** | 3.104 (1.067-9.028) | 0.038 |
| **Grade, III vs. I/II** | 1.287 (0.318-5.196) | 0.723 |
| **Ki-67, >20% vs. ≤20%** | 2.623 (0.321-21.407) | 0.368 |
| **CT regimen, others vs. Taxane based** | 0.308 (0.035-2.700) | 0.288 |

^a^Unknowns excluded.

Abbreviations: CI, confidence interval; RSI, radiosensitivity index; RR, radioresistance subtype; RS, radiosensitive subtype; FUSCC, Fudan University Shanghai Cancer Center; BLIS, basal-like and immune suppressed; CT, chemotherapy; RFS, Recurrence-free survival; HR, hazard ratio.

**Table S3. Multivariate analysis modeling with integrated BLIS-RR subtype and clinicopathologic variables for RFS in TNBC patients treated with RT.**

| **Variable^a^** | **RFS** | |
| --- | --- | --- |
|  | **HR (95% CI)** | ***P*** |
| **Integrated subtype, BLIS-RR vs. others** | 8.175 (1.939-34.454) | 0.004 |
| **Age (y), ≤40 vs. >40** | 2.609 (0.794-8.565) | 0.114 |
| **Tumor size, ≥3.5cm vs. <3.5cm** | 0.263 (0.030-2.277) | 0.225 |
| **Lymph nodes, >4 vs. ≤3** | 3.379 (1.208-9.454) | 0.020 |
| **Grade, III vs. I/II** | 0.349 (0.103-1.176) | 0.090 |
| **Ki-67, >20% vs. ≤20%** | 3.846 (0.461-32.048) | 0.213 |
| **CT regimen, others vs. Taxane based** | 0.322 (0.039-2.606) | 0.288 |

^a^Unknowns excluded.

Abbreviations: CI, confidence interval; BLIS, basal-like and immune suppressed; RR, radioresistance subtype; CT, chemotherapy; RFS, Recurrence-free survival; HR, hazard ratio.

**Table S4. Multivariate analysis modeling with integrated BLIS-RR subtype, *SERPINB5,* and clinicopathologic variables for RFS in TNBC patients treated with RT.**

| **Variable^a^** | **RFS** | |
| --- | --- | --- |
|  | **HR (95% CI)** | ***P*** |
| **Integrated subtype, BLIS-RR vs. others** | 4.648 (1.045-20.668) | 0.044 |
| ***SERPINB5*, high vs. low** | 8.747 (1.972-38.785) | 0.004 |
| **Age (y), continuous** | 2.422 (0.704-8.335) | 0.161 |
| **Tumor size, ≥4cm vs. <4cm** | 2.412 (0.217-26.792) | 0.473 |
| **Lymph nodes, >4 vs. ≤3** | 3.798 (1.199-12.032) | 0.023 |
| **Grade, III vs. I/II** | 0.307 (0.081-1.165) | 0.083 |
| **Ki-67, >20% vs. ≤20%** | 10.926 (1.118-106.771) | 0.040 |
| **LVI, positive vs. negative** | 2.511 (0.296-21.315) | 0.399 |
| **CT regimen, others vs. Taxane based** | 0.436 (0.053-3.556) | 0.436 |

^a^Unknowns excluded.

Abbreviations: CI, confidence interval; BLIS, basal-like and immune suppressed; RR, radioresistance subtype; CT, chemotherapy; LVI, lymphovascular invasion; RFS, Recurrence-free survival; HR, hazard ratio.

**Table S5. Multivariate analysis modeling with *SERPINB5* and clinicopathologic variables for OS in TNBC patients of TCGA datasets**

| **Variable** | **RFS** | |
| --- | --- | --- |
|  | **HR (95% CI)** | ***P*** |
| ***SERPINB5*, high vs. low** | 8.910(1.211-65.538) | 0.032 |
| **RSI-subtype, RR vs. RS** | 0.617 (0.139-2.730) | 0.525 |
| **Age (y), ≤50 vs. >50** | 6.699 (1.403-31.973) | 0.017 |
| **Tumor size, ≥5.1cm vs. <5cm** | 370.105 (23.507-5826.935) | 0.000 |
| **Lymph nodes, continuous** | 1.889 (1.342-2.659) | 0.000 |
| **Histological type, others vs. IDC/ILC** | 3.893 (0.673-22.515) | 0.129 |
| **Surgery, lumpectomy vs. mastectomy** | 3.462 (0.816-14.686) | 0.092 |
| **CT regimen, no vs. yes** | 8.481 (1.421-50.632) | 0.019 |

Abbreviations: CI, confidence interval; RSI, radiosensitivity index; RR, radioresistance subtype; RS, radiosensitive subtype;IDC, Infiltrating Ductal Carcinoma; ILC, Infiltrating Lobular Carcinoma; CT, chemotherapy; OS, overall survival; HR, hazard ratio.

**Table S6. Radiosensitivity parameters of HCC1937 and MDA-MB-231 cells in each group (mean ± SD).**

| **Group** | **N (biological replicates)** | **D_0_ (Gy)** | **D_q_ (Gy)** | **SF_2_** | **SER** |
| --- | --- | --- | --- | --- | --- |
| HCC1937-SiNC | 3 | 1.10 ± 0.10 | 0.67 ± 0.16 | 0.61 ± 0.05 | - |
| HCC1937-SiSERPINB5 | 3 | 0.96 ± 0.14 | 0.52 ± 0.18 | 0.60 ± 0.06 | 1.15 ± 0.07 |
| MDA-MB-231-SiNC | 3 | 2.40 ± 0.10 | 2.30 ± 0.06 | 0.79 ± 0.06 | - |
| MDA-MB-231-SiSERPINB5 | 3 | 1.96 ± 0.17 | 1.64 ± 0.30 | 0.76 ± 0.01 | 1.23 ± 0.06 |

**Table S7. Primers for qPCR validation**

| Gene | Primer | |
| --- | --- | --- |
|  | Forward | Reverse |
| SERPINB5 | CGAAGAGACCGTATGCAAAGG | CTGGTCGTTCACACTGTTGTC |
| MUC16 | GTCAGCGAGGAGCCATTCA | TCTCAGCACCGTTCTTCACA |
| RGS4 | GAGAGTGAGCCAAGAGGAAGTC | AGGAAGCGGCGGTAGGAAT |
| CXCL8 | TCTCTTGGCAGCCTTCCTG | GGTCCACTCTCAATCACTCTCA |
| AR | GACCTGCCTGATCTGTGGAG | CGAAGACGACAAGATGGACAAT |
| SCGB2A2 | TGGCTGCCCCTTATTGGAGA | TGGCATTGTCGTCTATGAACTCT |
| PIK3CG | TGGAATAGGCGACAGACACAA | TCAGCATCATGGAGAACAGGAT |
| IL7R | CCCTCGTGGAGGTAAAGTGC | CCTTCCCGATAGACGACACTC |

**Table S8. siRNA information**

| Name | Sequence |
| --- | --- |
| siSERPINB5_001 | \| GAGACCGTATGCAAAGGAA \| \| --- \| |
| siSERPINB5_002 | GTGGCCCTATCAAATGTTA |
| siSERPINB5_003 | GCACAAGGATGAATTGAAT |
